# Non-digestible stachyose promotes bioavailability of genistein through inhibiting intestinal degradation and first-pass metabolism of genistein in mice

**DOI:** 10.1080/16546628.2017.1369343

**Published:** 2017-08-30

**Authors:** Yalong Lu, Dehui Lin, Wenfeng Li, Xingbin Yang

**Affiliations:** ^a^ Shaanxi Engineering Laboratory for Food Green Processing and Safety Control, and Key Laboratory of Ministry of Education for Medicinal Resource and Natural Pharmaceutical Chemistry, College of Food Engineering and Nutritional Science, Shaanxi Normal University, Xi’an, China; ^b^ Key Laboratory of Chongqing Municipality for Protection and Utility of Unique Plant Resources in the Wulingshan Region, Life Science and Technology Institute, Yangtze Normal University, Chongqing, China

**Keywords:** Stachyose, genistein, absorption, phase II enzymes, efflux transporters

## Abstract

This study was designed to explore the molecular mechanism of stachyose in enhancing the gastrointestinal stability and absorption of soybean genistein in mice. Male Kunming mice in each group (n = 8) were administered by intragastric gavage with saline, stachyose (250 mg/kg·bw), genistein (100 mg/kg·bw), and stachyose (50, 250, and 500 mg/kg·bw) together with genistein (100 mg/kg·bw) for 4 consecutive weeks, respectively, and then their urine, feces, blood, gut, and liver were collected. UPLC-qTOF/MS analysis showed that levels of genistein and its metabolites (dihydrogenistein, genistein 7-sulfate sodium salt, genistein 4’-β-D-glucuronide, and genistein 7-β-D-glucuronide) in serum and urine were increased with an increase in stachyose dosages in mice. Furthermore, the feces level of genistein aglycone was also elevated by co-treatment of stachyose with genistein. However, the feces concentration of dihydrogenistein, a characteristic metabolite of genistein by gut microorganism, was decreased by stachyose administration in a dose-dependent manner. Additionally, the simultaneous administration with stachyose and genistein in mice could decrease intestinal SULT, UGT, P-gp, and MRP1 expression, relative to the treatment with individual stachyose or genistein. These results demonstrate that stachyose-mediated inhibition against the intestinal degradation of genistein and expression of phase II enzymes and efflux transporters can largely contribute to the elevated bioavailability of soybean genistein.

## Introduction

In the past few decades, consumers have paid more and more attention to health, and the overall quality of life has increased, which boosts the demand of natural healthy foods [1]. Soy as a kind of functional and traditional food has aroused great interest in researchers due to the major source of flavonoids [2]. In soybean flavonoids, genistein is a natural isoflavone in the family of secondary plant metabolites, which possesses well-known preventive activity in breast and prostate cancer, cardiovascular diseases, and postmenopausal problems [3]. However, the low water solubility and intestinal permeability may lead to its poor oral bioavailability [4].

In recent years, a few studies have documented that some non-digestible carbohydrates, such as fructo-oligosaccharides, can enhance bioavailability of genistein, daidzein, αG-rutin, and α-glucosyl-isoquercitrin [5–7]. Similarly, our previous studies also indicated that soy stachyose as raffinose family oligosaccharide could increase absorption of tea polyphenols or soy genistein and hepatic protective effects in mice [8,9]. Interestingly, some oligosaccharides were approved to strongly suppress the bacterial degradation of quercetin aglycone, thus contributing to the increased bioavailability of quercetin glycoside [10]. It is worth noting that stachyose is a kind of natural prebiotic and its consumption was able to favorably modulate the composition of the intestinal microbiota in humans and animals [11]. For this reason, it is necessary to evaluate the hypothesis that prebiotic stachyose might inhibit intestinal degradation of genistein via modulating the composition of the intestinal flora.

It is interesting to note that flavonoids easily undergo first-pass metabolism catalyzed by intestinal and hepatic phase II metabolic enzymes, leading to low bioavailability of flavonoids [12,13]. In addition, it was reported that long-term treatment of genistein could induce the related protein expression of first-pass metabolism in mice [14,15] and the activities of phase II metabolic enzymes in mice could be mediated by oligosaccharides [13,16,17]. Moreover, some non-digestible carbohydrates, such as hyaluronan oligosaccharides and astragalus polysaccharides, were shown to inhibit P-gp, MRP1, and MRP2 expression, thereby contributing to an increase in intracellular concentration of food ingredients or drugs [18,19]. Accordingly, we suppose that stachyose promoting absorption of genistein may be closely related to inhibition of first-pass metabolism of genistein in the small intestine.

Therefore, the objective of the present study is to provide insights into the mechanism of stachyose to enhance absorption of genistein. UPLC-qTOF/MS was employed to quantify the levels of naturally occurring genistein and its metabolites (dihydrogenistein, genistein 7-sulfate, genistein 4’-β-D-glucuronide, genistein 7-β-D-glucuronide) in the serum, urine, and feces of the tested mice with consumption of genistein or its combination with stachyose. Furthermore, we also measured the expression of phase II metabolic enzymes (SULT and UGT) and efflux transporters (P-gp, MRP1, and MRP2) in the small and large intestine, as well as efflux transporters (UGT and SULT) in the liver. This study tests the hypothesis that stachyose may improve bioavailability of genistein via inhibiting intestinal degradation and first-pass metabolism of soy genistein.

## Materials and methods

### Materials and chemicals

**Figure 1.**
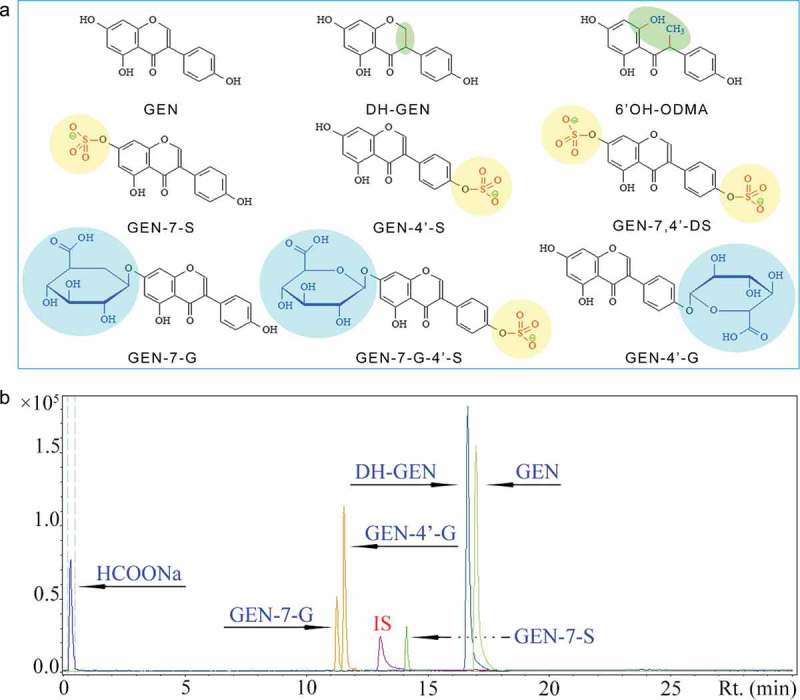
Chemical structure of genistein (GEN) and its metabolites in vivo (**a**). UPLC-qTOF/MS chromatogram of a standard substance mixture of analytes in urine, and the extracted ion chromatograms of single MRM traces are displayed (**b**).

The stachyose (pure>80%) was extracted from soybean residues in our laboratory [8]. Genistein (pure>98%) was isolated from soybean and obtained from Yongyi Biology Co. Ltd (Xi’an, China). Pure standards of genistein (GEN, 98%) and its metabolites dihydrogenistein (DH-GEN, 96%), genistein 7-sulfate sodium salt (GEN-7-S, 96%), genistein 4’-β-D-glucuronide (GEN-4’-G, 98%), genistein 7-β-D-glucuronide (GEN-7-G, 95%), and internal standard (2r, 3r)-dihydroquercetin (98%) were purchased from Toronto Research Chemicals Inc. (Toronto, Canada), and their chemical structural formula is shown in Figure 1(a). ELISA (enzyme-linked immuno sorbent assay) kits of the P-glycoprotein (P-gp), multidrug resistance-associated protein-1 (MRP1), multidrug resistance-associated protein-2 (MRP2), uridinediphosphate-glucuronosyltransferase (UGT), and sulfotransferase (SULT) were purchased from Shanghai Enzyme-linked Biological Technology Co., Ltd (Shanghai, China). All other reagents and chemicals were the highest grade available.

### Animals and experiment design

Forty-eight male Kunming mice were purchased from the Experimental Animal Center of the Fourth Military Medical University (Xi’an, Shaanxi, China) and housed in stainless-steel cages with wire-mesh bottoms, and temperature (22 ± 2^°^C) and humidity (55–65%) were controlled in a 12-h light/dark cycle. All the mice had free access to tap water and were offered pellets of commercial chow for one week after arrival. The mice were randomly divided into six groups (n = 8). In the control group, the mice were administrated intragastrically (i.g.) with 1% CMC-Na solution (0.4 mL) once daily. In the stachyose and genistein groups, the mice were administrated with 250 mg/kg.bw stachyose in 1% CMC-Na solution (i.g., 0.4 mL) and 100 mg/kg·bw genistein in 1% CMC-Na solution (i.g., 0.4 mL) once daily, respectively. For both genistein and stachyose co-treated groups (GS50, GS250, GS500), the mice were administrated with 100 mg/kg·bw genistein and three different doses of stachyose (50, 250, and 500 mg/kg·bw) in 1% CMC-Na solution (i.g., 0.4 mL), respectively. During the administration period within four weeks, all the tested mice were allowed free access to tap water and chow. After the last administration, all the animals were fasted but allowed free access to water as usual for 12 h, and then the urine and feces of the mice were collected and immediately stored at −80^°^C. Finally, the animals were fully anesthetized by inhalation of isoflurane, and sacrificed to obtain blood, small and large intestines, and the livers, which were stored at −80^°^C. This animal study abided by the Committee on Care and Use of Laboratory Animals of the Fourth Military Medical University (XJYYLL-2015689), China.

### UPLC-qTOF/MS analysis of genistein and its metabolites in the serum, urine and feces

The serum (150 μL) was added to a 1.5 mL centrifuge tube with methanol (1000 uL) and an internal standard of (2r, 3r)-dihydroquercetin (20 μL, 0.3 mg/mL). Supernatant of urine was collected after centrifugation at 3000*g* for 15 min. Feces (2.5 g) were also homogenized with 20 mL normal saline (w/v), and centrifuged at 3000*g* for 15 min and the supernatant was collected for measurement of genistein and its metabolites. The supernatants of urine (200 μL) and feces (100 μL) solution were added to 1.5 mL centrifuge tube with methanol (1000 μL) and an internal standard of (2r, 3r)-dihydroquercetin (20 μL, 0.3 mg/mL), respectively, and then each sample was centrifuged and filtered with a 0.22 um PVDF membrane for ultra-high liquid chromatography quadrupole time of flight mass spectrometry (UPLC-qTOF/MS) analysis. Gnistein and its metabolites were identified and quantified by UPLC-qTOF/MS using an electric spray ionization (ESI) interface (UltiMate 3000 High Speed UPLC System, Thermo Fisher Scientific Inc., MA, USA). The temperature of the capillary heater and the vaporization heater were maintained at 220°C and 450°C, respectively. UPLC-qTOF/MS analysis was carried out in scan mode from 50 to 1200 (m/z) and in selected ion monitoring mode of negative (m/z) 303.0 for (2r, 3r)-dihydroquercetin (internal standard), (m/z) 268.9 for genistein, (m/z) 270.9 for dihydrogenistein, (m/z) 348.9 for genistein 7-sulfate, and (m/z) 445.0 for genistein 4’-β-D-glucuronide and genistein 7-*β*-D-Glucuronide. The Thermo UPLC system was fitted with a 2.2 μm C_18_ column (Acclaim^TM^ RSLC, 3.0 × 100 mm, Thermo Co. Ltd., Milford, MA, USA) set at 25^○^C. Nitrogen was used as the nebulizer (1.2 mL/min) and collision gas (8 mL/min). Solvent A was water with a 40 mM ammonium formate and solvent B was acetonitrile. The flow rate of the mobile phase was 0.2 mL/min, and the mobile phase was programmed as follows: 95–70% A (v/v) from 0 to 5 min, 70–40% A (v/v) from 5 to10 min, 40–10% A (v/v) from 10 to 15 min, 10% A (v/v) from 15 to 18 min, 10–95% A (v/v) from 18 to 25 min, 95% A (v/v) from 25 to 30 min. The total run time was 30 min, and the column equilibration time between each run was 5 min.

### ELISA analysis of the intestine and liver of mice

Intestinal tissue (0.3 g) or liver tissue (0.3 g) was homogenized with 2.7 mL normal saline and centrifuged at 3000*g* for 10min. After getting the supernatant, the levels of UGT, SULT P-gp, MRP1, and MRP2 in small and large intestinal tissue, and UGT and SULT expression in the liver tissue was detected by commercial ELISA kits according to the relevant manufacturer’s instruction.

### Statistical analysis

All results were presented as the means ± SD and analyzed using ANOVE. All the statistical analyses were carried out using SPSS 20 (IBM). Values were considered significant when *p*<0.05. The heat-map was drawn using HemI 1.0.1.

## Results

### Stachyose dose-dependently enhanced bioavailability of genistein in mice

**Figure 2.**
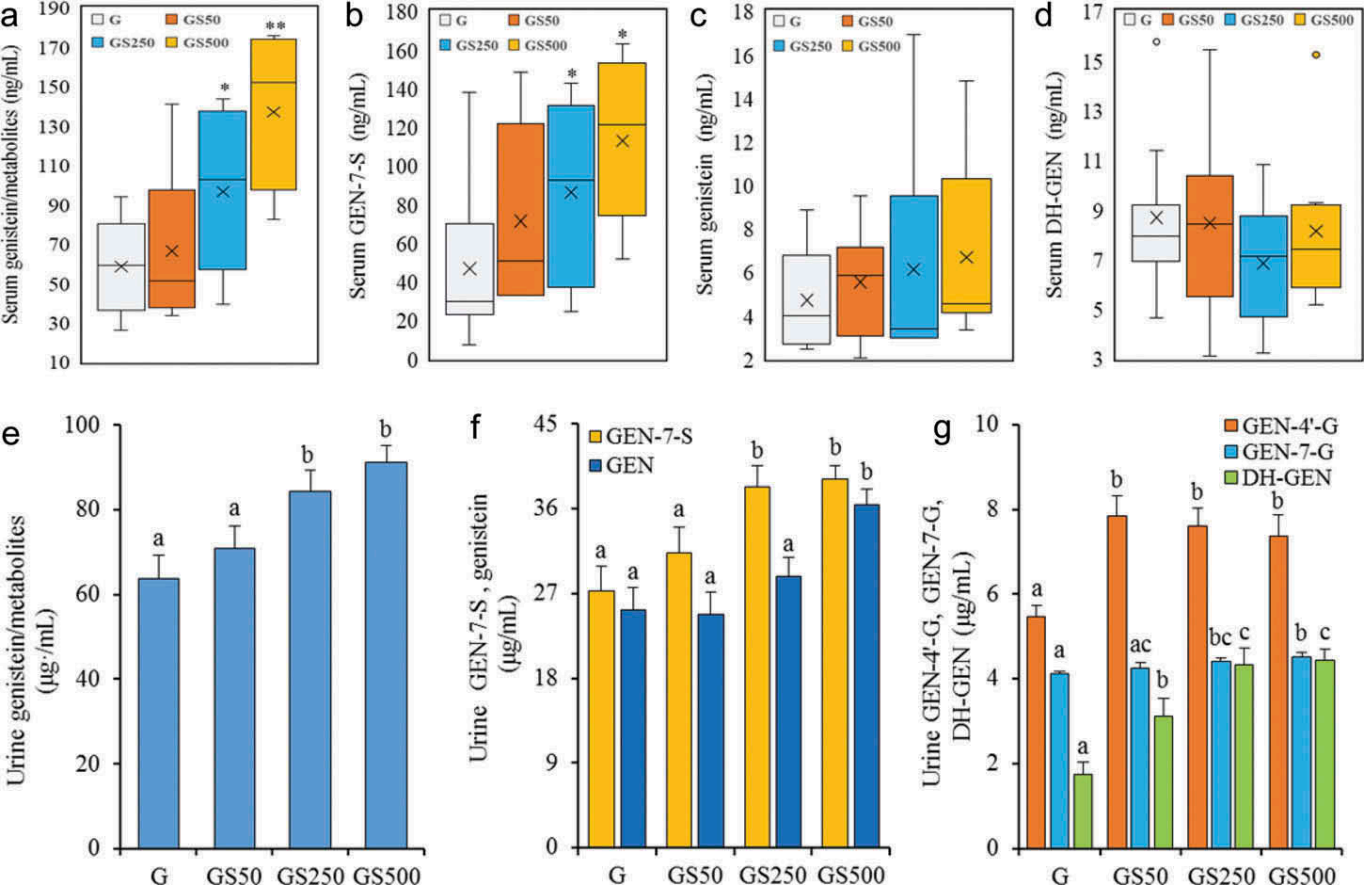
Treatments of stachyose at 50, 250 and 500 mg/kg·bw for 4 weeks increased serum concentrations of total genistein and its metabolites (total genitein/metabolites, (**a**), genistein 7-sulfate sodium salt (GEN-7-S, **b**), and genistein (GEN, **c**), dihydrogenistein (DH-GEN, **d**), respectively, and urinary concentrations of total genistein and its metabolites (total genitein/metabolites, **e**), and genistein 7-sulfate sodium salt and genistein (GEN-7-S and GEN, **f**) and genistein 4’-β-D-glucuronide, genistein 7-sulfate sodium salt, and dihydrogenistein (GEN-4’-G, GEN-7-G and DH-GEN, **g**), respectively. Values of biochemical parameters are expressed as means ± SD of 8 mice in each group. ^a^^–^^c^ Mean values within a figure with different alphabetical letters denote significant differences (*p<*0.05) among all the groups.

To evaluate the effects of stachyose on the absorption of genistein, the mice were simultaneously administrated with genistein at 100 mg/kg·bw and different doses of stachyose at 0, 50, 250, and 500 mg/kg·bw for 4 weeks, respectively. The serum, urine, and feces concentrations of genistein and its metabolites (GEN-7-S, GEN-4’-G, GEN-7-G, and DH-GEN, Figure 1(a)) in the mice were identified and quantified by UPLC-qTOF/MS, and MRM chromatogram of standards mixture was obtained by UPLC-qTOF/MS (Figure 1(b)). In order to investigate the accuracy of the method, recovery experiments were performed. Known amounts of each standard substance solute were added to the sample detected, and the resulting spiked sample was subjected to the entire analytical sequence. Each solute was spiked at a close concentration with the sample and recoveries were calculated based on the difference between the total amount determined in the spiked samples and the amount observed in the non-spiked samples. All analyses were carried out in triplicate. The results show that the recoveries of all the genistein and its metabolites ranged between 95.56% and 104.02%. Furthermore, the method precision was determined by measuring repeatability (intra-day variation coefficient) and intermediate precision (inter-day variation coefficient) of peak area for each tested object. The precision of method was calculated as the variation coefficient (CV) for five successive injections of the same sample. The results showed that the intra-day CV was less than 2.01% for the peak areas, and the inter-day CV values were less than 2.39% for the for the peak areas. These results indicated that the conditions used in the quantitative determination of genistein and its metabolites in the serum, urine, and feces were appropriate. As shown in Figure 2(a), simultaneous ingestion of stachyose at 50 mg/kg·bw (*p*>0.05), 250 mg/kg·bw (*p*<0.05) and 500 mg/kg·bw (*p*<0.05) in mice could enhance the serum concentrations of total genistein and its metabolites which are calculated as the sum of genistein, its sulfated (GEN-7-S) and hydrogenated (DH-GEN) metabolites in a dose-dependent manner. Meanwhile, the methylated or glucuronidated metabolites of genistein were not detected in serum by UPLC-qTOF/MS, and the levels of serum GEN-7-S as the main metabolite of genistein sulfation in GS250 and GS500 treated mice were significant higher by 2- and 3-fold (*p*<0.05) than that in individual genistein-fed mice, respectively (Figure 2(b)), and the effect was dependent on the dosages. It was also found that the serum-free genistein aglycone concentration was slightly increased by the combined treatment with stachyose (*p*>0.05, Figure 2(c)), and the levels of serum dihydrogenistein (DH-GEN), a characteristic metabolite of genistein by gut microorganism, showed no significant change in co-treated mice (*p*>0.05) when compared with only genistein-treated mice (Figure 2(d)). These results demonstrate that the combination of stachyose with genistein could strongly increase bioavailability of genistein by improving the serum GEN-7-S metabolism of mice.

Urine is favored as a reliable biomarker to assess genistein bioavailability because of its high secretion level compared to plasma or the other body fluids, and therefore urine was used to extrapolate systemic exposure of genistein metabolism in this work [20]. As shown in Figure 2(e), the co-treatment of stachyose at 250 and 500 mg/kg·bw remarkably caused the 32.2% and 42.7% increases in 12-h cumulative uninary excretion of total genistein and its metabolites including sulfated (GEN-7-S) and glucuronidated (GEN-4’-G and GEN-7-G), and hydrogenated (DH-GEN) metabolites of mice in comparison with single genistein treatment, respectively (*p*<0.05). Meanwhile, genistein showed the highest excretion and its levels were obviously enhanced by combined administration compared to individual genistein treatment in a dose-dependent manner, and the GEN-7-S levels were also increased by supply of stachyose, where the urinary level of GEN-7-S was elevated by 14.9% (*p*>0.05), 40.5% (*p*<0.05) and 43.7% (*p*<0.05), relative to that of individual genistein-treated mice, respectively (Figure 2(f)). In comparison with the metabolites of circulating genistein in the blood, two glucuronidated genisteins (GEN-4’-G and GEN-7-G) were detected in urine, and urinary GEN-4’-G as the main glucuronidated metabolite in the co-treated mice presented noteworthy elevation (*p*<0.05) when compared to that of individual genistein-treated mice (Figure 2(g)). Furthermore, DH-GEN, which was the characteristic metabolite of genistein by gut microorganism, were dose-dependently increased from 1.75 μg/mL of single administration of genistein group to 3.11 μg/mL (*p*>0.05), 4.32 μg/mL (*p*<0.05) and 4.44 μg/mL (*p*<0.05) of the co-treated group (Figure 2(g)). These data clearly show that stachyose can increase genistein and its metabolites levels in mouse serum and urine, further indicating that stachyose has capacity to enhance the bioavailability of genistein in vivo.

### Stachyose improve the stability of genistein in mouse intestine

**Figure 3.**
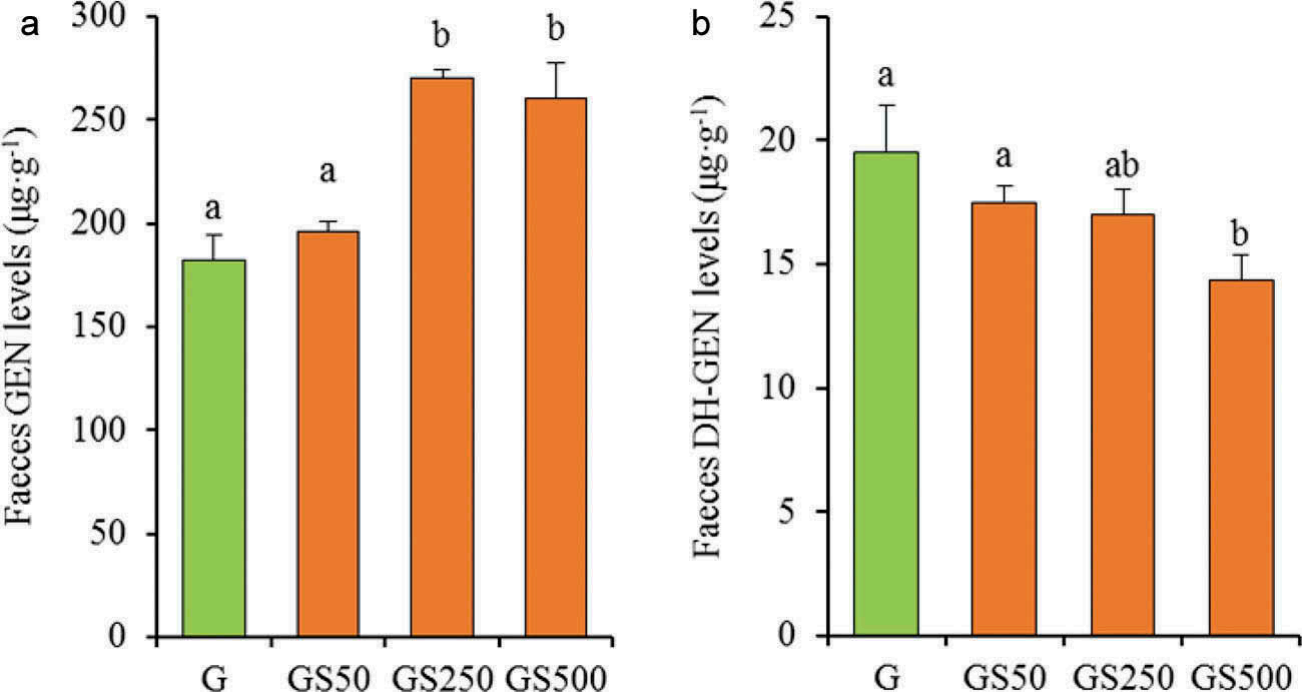
The levels of Genistein (GEN, **a**) and Dihydrogenistein (DH-GEN, **b**) in faeces by administration of different dose of stachyose. The letters (a–b) denote that the mean values with different letters are significantly different (*p*<0.05).

It is widely acknowledged that the degradation of flavonoid in the gut is the important factor influencing its bioavailability [10]. Herein, we employed UPLC-qTOF/MS to determinate genistein aglycone and its gut microorganism-based characteristic metabolite (DH-GEN) in feces in order to indirectly evaluate the stability of genistein in the gut. As described in Figure 3(a), the genistein excretion of feces after ingestion of stachyose was significantly improved by 1.2-, 1.5-, and 1.7-fold (GS50, GS250, and GS500, *p*>0.05, *p*<0.05 and *p*<0.05) when compared with application of individual genistein, respectively, suggesting that simultaneous ingestion of stachyose improved the stability of genistein in mouse intestine. Furthermore, the combination treatments were found to remarkably reduce the urinary excretion of DH-GEN, a characteristic product of genistein degradation from intestinal microflora [21], and this effect was dose-dependent from 18.38 μg/mL in the individual genistein treated mice to 17.5 μg/mL, 17.1 μg/mL, and 14.1 μg/mL (GS50, GS250, and GS500, Figure 3(b)). These results indicated that the degradation of genistein in the intestine was suppressed by treatment of stachyose.

### Stachyose inhibits the expression of phase II enzymes in small intestine

**Figure 4.**
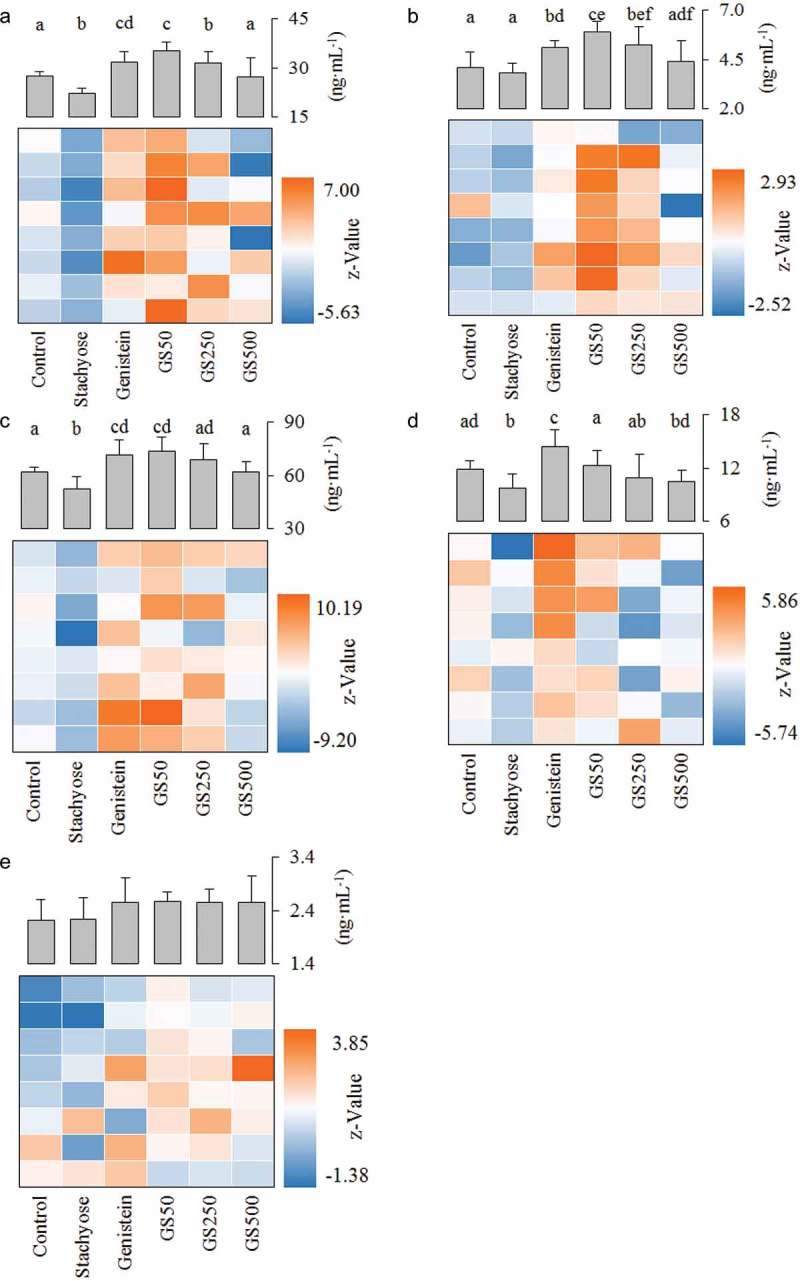
Effects of treatment of stachyose at different dose for 4 weeks on small intestinal UGT (**a**), SULT (**b**), P-gp (**c**), MRP1 (**d**), and MRP2 (**e**) levels, respectively. The color of the heat map reflects the Z-value of the concentration of protein. Z-value = (X*_i_*-X*_control_*)/SD*_control_*, where X*_i_* is the level of a random metabolite in serum, X*_control_* is the average value of the serum level of normal diet-fed mice, and SD*_control_* is the standard deviation of the metabolite level in the serum of normal diet-fed mice. The letters (a–f) denote that the mean values with different letters are significantly different from one another (*p*<0.05).

The phase II enzymes UGT and SULT are significant metabolic pathways for numerous endo- and xeno-biotics in the enterocytes, which are also known to interact with the ingested flavonoids and accelerate efflux transportation of flavonoids to reduce bioavailability of flavonoids [12, 22]. As shown in Figure 4(a, b), treatment of stachyose alone was able to inhibit the expression of small intestinal UGT and SULT, respectively (*p*<0.05), while treatment of individual genistein caused a severe rise in UGT and SULT, compared to that in the control mice, respectively (*p*<0.05). Interestingly, the UGT expression in the small intestine was remarkably decreased by 10.4% and 14.8% when the dose of the co-treated stachyose was increased to 250 and 500 mg/kg·bw in mice compared to individual genistein treatment, respectively (Figure 4(a)). Similarly, this combination also significantly reduced the levels of SULT from 5.92 of individual genistein-treated mice to 4.37 ng/mL of the high-dose stachyose-treated mice (*p*<0.05, Figure 4(b)). These results showed that non-digestible stachyose could inhibit expression of intestinal phase II enzymes to improve the bioavailability of genistein in mice.

### Stachyose reduces the expression of efflux transporters in small intestine

P-gp, a well-known efflux pump responsible for multiple drug resistance, plays a major role in the cells to transport genistein from the intra-cell to the outside [23,24]. As represented in Figure 4(c), the small intestinal P-gp level in mice treated with stachyose alone showed a significant decrease (*p*<0.05), but an independent treatment with genistein showed a significant increase (*p*<0.05), relative to the intestinal P-gp expression in the untreated control group. Interestingly, the co-administration could dose-dependently reduce the P-gp level in the small intestine, compared to single genistein treatment (Figure 4(c)). In addition, MRP1 and MRP2 are also two major membrane efflux transporters of phase II metabolites of genistein [25, 26]. Figure 4(d) revealed that application of stachyose caused a sharp decrease in MRP1 level (*p*<0.05) and administration of genistein induced a remarkable increase of MRP1 level (*p*<0.05) as compared to that in the control group. Interestingly, the co-administration of both stachyose and genistein was able to decrease the MRP1 expression in intestine tissue by 15.07% (*p*<0.05), 24.42% (*p*<0.05), and 27.43% (*p*<0.05), relative to only genistein treatment, respectively (Figure 4(d)). However, there was no significant change in small intestinal MRP2 expression of the mice treated with stachyose, genistein, or in combination (Figure 4(e)). These data indicate that simultaneous administration of genistein with stachyose could inhibit intestinal P-gp and MRP1 expressions, contributing to the elevation of genistein bioavailability in mice.

### Effects of stachyose/genistein on first-pass metabolism in large intestine and liver

**Figure 5.**
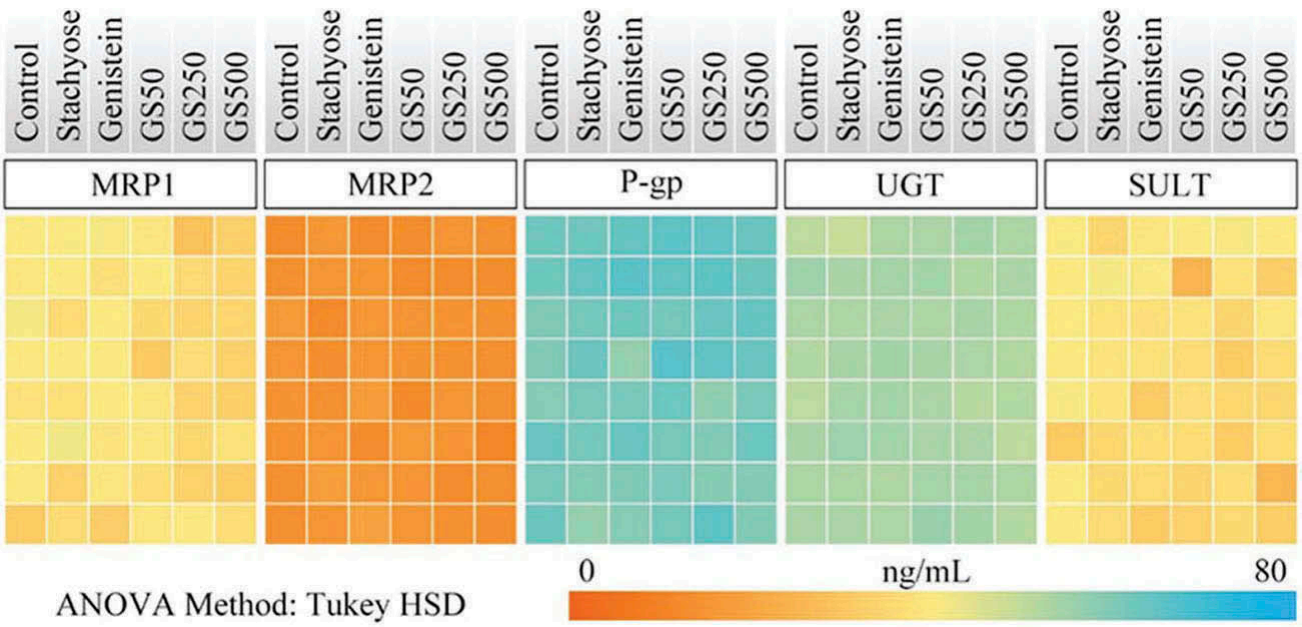
A heat map analysis was performed to describe the expression of phase II metabolic enzymes and transport proteins in the large intestine. The color of the heat map directly reflects the concentration of protein. The more similar color represented the smaller difference of the concentration.

The large intestine is an important site for absorption of isoflavonoids [27]. In the present study, the MRP1, MRP2, P-gp, UGT, and SULT levels of the large intestinal tissues in mice were evaluated by ELISA assay and the results are shown in Figure 5. The ANOVE analysis indicated that stachyose could not influence expressions of MRP1, MRP2, P-gp, UGT, or SULT in the large intestine of mice, which suggested that stachyose had no effect for the expression of phase II enzymes and efflux transporters in the large intestine.

**Figure 6.**
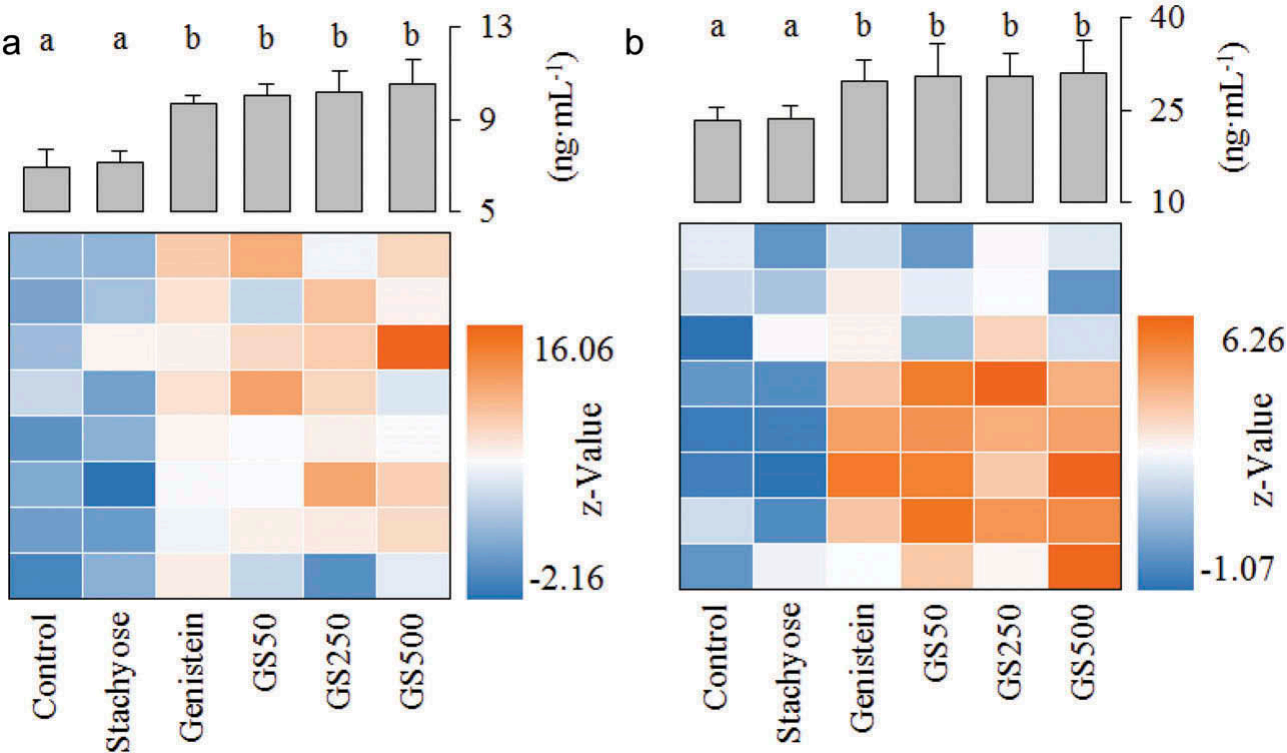
Effects of stachyose and genistein on hepatic UGT (**a**) and SULT (**b**) of mice, respectively. The letters (a–b) denote that the mean values with different letters are significantly different from one another (*p*<0.05).

As is well known, the liver is also an important target for phase II glucuronidation and sulfation metabolism of flavonoids, which is mainly SULT and UGT [22,28]. Unexpectedly, treatment with stachyose alone could not affect (*p*>0.05) SULT and UGT expression levels in mouse liver as compared to that of normal mice (Figure 6). Although treatment of genistein alone could powerfully increase hepatic SULT and UGT levels by 35.74 and 27.55%, relative to that in normal mice (*p*<0.05), the combination of genistein with stachyose did not display statistical differences in hepatic UGT and SULT expression in comparison with individual genistein treatment, respectively (*p*>0.05). These results indicated that the phase II enzymes of the intestine, not the liver, were the target of stachyose for improving the bioavailability of genistein.

## Discussion

Our works demonstrated that the combined treatment with stachyose and genistein could obviously enhance bioavailability of genistein (Figure 2). It was suggested that the possible potential mechanism was associated with the inhibition of non-digestible oligosaccharides against the bacterial degradation of quercetin aglycone in the intestine [10], but this effect has not yet been confirmed by analysis of the microbial characteristic metabolites of flavonoids. In the present study, the DH-GEN was found to be a characteristic metabolite of genistein degraded by intestinal microflora, and its feces excretion of the tested mice in the co-treatment groups was also decreased with the increased doses of simultaneous ingested stachyose (Figure 3). In contrast, the feces levels of genistein aglycone in co-administrated mice were increased in a dose-dependent manner, suggesting that stachyose intake improved the stability of genistein in the gastrointestinal tract. Our previous investigation has also indicated that stachyose as a kind of prebiotic could regulate intestinal bacteria [11], and therefore we speculated that the simultaneous ingestion of stachyose in mice might inhibit bacterial degradation of genistein in the gut, resulting in the enhance in the bioavailability of genistein in vivo. Furthermore, in comparison with treatment of individual genistein, the present study showed that urinary excretion of DH-GEN as gut microflora-based metabolite of genistein displayed a dose-dependent increase in the mice treated together with stachyose (Figure 2), strongly indicating that intestinal degradation inhibition of genistein glycoside was not the only reason for promoting the bioavailability of genistein by stachyose.

Numerous studies have confirmed that genistein easily undergoes phase II biotransformation mainly including sulfonation and glucuronidation in intestinal epithelial cells, which may contribute to the first-pass metabolism of genistein, resulting in its low bioavailability [21,29,30]. Previous reports [14] show that UGT and SULT expression in the intestine could be elevated by single long-term treatment with genistein, leading to the low bioavailability of genistein, which was also confirmed in our study. Our study also demonstrated that small intestinal UGT and SULT levels were inhibited with an increase of stachyose dosages in the co-treated mice (Figure 4), indicating that the phase II metabolic transformation of genistein was suppressed by simultaneous supplementation with stachyose in mice, which might be beneficial to an increase in absorption of genistein.

Recent researches have shown that the phase II metabolites of genistein were not allowed to efflux among cells by a simple diffused way, which is a key step for modifying bioavailability of flavonoids [25,28,31]. Instead, they highly depend on the activity of membrane bound ATP binding cassette transport proteins (such as P-gp, MRP1, and MRP2) to over the intestinal epithelium [25,31]. A previous study has reported that efflux transporters' (P-gp, MRP1, and MRP2) expression of cancer cells is improved in a dose-dependent manner by genistein treatment for a long time [32], which would strengthen the first-pass metabolism of genistein (Figure 4). Interestingly, in the present study, co-treatment of stachyose could decrease the levels of P-gp, MRP1, and MRP2 in the small intestine with the increase of treatment doses when the mice were co-administrated for four consecutive weeks (Figure 4), suggesting that stachyose could inhibit efflux transportation of phase II metabolites of genistein in the intestine of mice, which contributed to promote bioavailability of genistein. In other words, co-administration of stachyose strongly inhibited the efflux transportation of phase II metabolites of genistein, leading to an accumulation phase II metabolites of genistein in serum and urine. Furthermore, simultaneous ingestion of stachyose could also enhance the absorption of the DH-GEN according to the levels of efflux transporters in the intestine of mice (Figure 4), and this might be a reason that the combined treatment reduced feces DH-GEN level but increased DH-GEN level in the urine of mice.

Matsukawa et al. have shown that the large intestine is one of the crucial target organs for non-digestible oligosaccharides to promote bioavailability of flavonoid glycosides [10]. Some other studies have also reported that the large intestine is a main position to absorb genistein [20]. However, our present research found that genistein, stachyose, or combined treatment did not influence the expression of phase II metabolic enzymes and efflux transporters in the large intestine of mice (Figure 5). Accordingly, it is suggested that the UGT and SULT of the large intestine is not the target spot. It has been generally reported that the diet flavonoids, which are absorbed in small intestine epithelial cell and transported to blood circulation, are further metabolized in the liver by phase II metabolic enzymes [22,33]. The present research suggested that the levels of SULT and UGT were increased when the mice were administrated with genistein for four consecutive weeks (Figure 6), which is highly consistent with previous studies [14]. However, co-treatment of stachyose and genistein did not change the levels of phase II metabolic enzymes in the liver of mice (Figure 6). For these reasons, all the findings in this work strongly indicated a potential molecular mechanism that non-digestible stachyose promotes bioavailability of genistein through improving the stability of genistein in the gut and inhibiting small intestinal degradation and first-pass metabolism of genistein in mice. It was also suggested that stachyose promoted the absorption of genistein aglycone which was related to the improved physiological activity of genistein, and it might be one of the important factors for the capacity of stachyose to strengthen the hepatoprotective activity of genistein.

In conclusion, the present results clearly showed that stachyose significantly enhanced the absorption of genistein in mice. Also, it was demonstrated for the first time that feeding with stachyose strongly inhibited the intestinal degradation of genistein and expression of the phase II enzymes and efflux transporters in the intestine, which was a novel mechanism for the promotion of genistein absorption by stachyose in the intestine of the mouse. All these findings demonstrate the mechanism that non-digestible oligosaccharide enhances bioavailability of soybean genistein, and provides a perfect theoretical basis for the application of stachyose and genistein in the food industry.
